# The Passive Film Growth Mechanism of New Corrosion-Resistant Steel Rebar in Simulated Concrete Pore Solution: Nanometer Structure and Electrochemical Study

**DOI:** 10.3390/ma10040412

**Published:** 2017-04-14

**Authors:** Jin-yang Jiang, Danqian Wang, Hong-yan Chu, Han Ma, Yao Liu, Yun Gao, Jinjie Shi, Wei Sun

**Affiliations:** 1 School of Materials Science and Engineering, Southeast University, Nanjing 211189, China; wonderbaba@126.com (D.W.); chuhongyan87@126.com (H.-y.C.); liuyao0629@126.com (Y.L.); gaoyun3888@126.com (Y.G.); jinjies@126.com (J.S.); sunwei@seu.edu.cn (W.S.); 2 Jiangsu Key Laboratory of Construction Materials, Nanjing 211189, China; 3 Research Institute of Jiangsu ShaSteel Iron and Steel, Zhangjiagang 215625, China; mahan-iris@shasteel.cn

**Keywords:** corrosion-resistant steel rebar, passive film, TEM

## Abstract

An elaborative study was carried out on the growth mechanism and properties of the passive film for a new kind of alloyed corrosion-resistant steel (CR steel). The passive film naturally formed in simulated concrete pore solutions (pH = 13.3). The corrosion resistance was evaluated by various methods including open circuit potential (OCP), linear polarization resistance (LPR) measurements, and electrochemical impedance spectroscopy (EIS). Meanwhile, the 2205 duplex stainless steel (SS steel) was evaluated for comparison. Moreover, the passive film with CR steel was studied by means of X-ray photoelectron spectroscopy (XPS), transmission electron microscopy (TEM), Atomic Force Microscope (AFM), and the Mott‑Schottky approach. The results showed that the excellent passivity of CR steel could be detected in a high alkaline environment. The grain boundaries between the fine passive film particles lead to increasing Cr oxide content in the later passivation stage. The filling of cation vacancies in the later passivation stage as well as the orderly crystalized inner layer contributed to the excellent corrosion resistance of CR steel. A passive film growth model for CR steel was proposed.

## 1. Introduction

The corrosion of steel is one of the most important factors for the damage of reinforced concrete structures. A protective passive film is usually formed on the steel surface in the high alkaline concrete environment. Carbonation and chloride induction are two main causes that induce steel corrosion. Therefore, the key solution to avoid corrosion problems is to enhance the passivity and anti‑chloride-corrosion ability of steel rebar [1,2]. In general, coated steel rebar is the main corrosion improvement method, such as epoxy‑coated steel rebar and alloyed steel rebar [3,4,5]. Coated steel rebar has been developed for decades. However, the suitability of the coating to the environment of fresh and hardening concrete is still debatable [6]. Moreover, the tiny defects that exist in the coating layer are the activated sites for pitting, which results in sudden devastating corrosion [7].

Stainless steel rebar has been used to solve corrosion problems in extremely severe environments such as marine environments for many decades [8]. The high corrosion resistance of stainless steel is attributed to the compact dual-layer passive film on the steel surface. The passive film can be influenced by the composition and microstructure of the steel substrate [9]. The descaled austenitic and duplex stainless steel (≥18% Cr and ≥8% Ni) is often used due to the high pitting resistance equivalency number (PREN) [10]. The use of ferritic stainless steels is recommended for mild environments, where carbonation is the only cause of attack [11]. The high alloy content of stainless steel leads to excellent corrosion resistance, but results in high cost and bad weldability [5]. With proper processing and metallographic microstructure, the low alloy content can also provide adequate corrosion resistance in high alkaline environments, which also leads to low cost and good weldability. Therefore, reinforcing steel with medium alloy content has drawn much attention recently. Typical examples include the ASTM (American Society for Testing and Materials) A1035 with 9% Cr and martensitic microstructure, and the Low‑Ni 200 grade austenitic stainless steels [12].

The passive film is the barrier that protects steel from corrosion, which can be influenced by the external environment as well as the steel substrate [13]. Although the passive film of stainless steel has been widely investigated [14], the relationship between the passive film and substrate properties such as composition and microstructure is still a matter of discussion. It was claimed that the surface film with Alloy 600 and Alloy 690 at the early stages of the oxidation process consisted of Cr_2_O_3_ and Ni^2+^ other than Fe^3+^/Fe^2+^ [15,16]. Machet et al. [15] pointed out that Cr_2_O_3_ would firstly nucleate and grow on the surface. Ziemniak et al. [17] noted that Cr and OH^−^ predominantly form Cr hydroxide at the alloy/solution interface due to the lower standard free energy of Cr(OH)_3_ than Cr_2_O_3_. Chen et al. [18] reported that nanocrystallization altered the nucleation mechanism of the passive film on the conventional rolled coarse crystalline (CC) 304ss, from being progressive to being instantaneous. Due to the distinct composition and microstructure, the passive film growth mechanism of CR steel has to be studied thoroughly. The objective of this work is to investigate the passive film growth mechanism and the effect of substrate composition of new 10% Cr alloyed corrosion-resistant steel (CR). An elaborative study is carried out on the composition, structure, and morphology of the passive film during the whole passivation period. Various methods are applied, such as electrochemical methods (open‑circuit potential, linear polarization resistance, electrochemical impedance spectroscopy, and Mott-Schottky curves), X-ray photoelectron spectroscopy (XPS), transmission electron microscopy (TEM), and atomic force microscopy (AFM).

## 2. Experimental Methods

### 2.1. Materials

The samples were made of discs of 10 mm thickness drilled from a rod of 16 mm diameter. The chemical composition of the new kind of alloyed corrosion-resistant steel (CR steel), duplex stainless steel (SS) 2205, and low-carbon steel (LC) rebar are shown in Table 1.

The drilled samples were ground and polished to 2.0 μm, which aimed to reduce the surface heterogeneity. Then, the samples were ultrasonically rinsed with distilled water and subjected to ultrasonic cleaning in acetone. The air dried samples were prepared and used for all the electrochemical corrosion experiments.

For conducting the corrosion tests in different chloride‑induced environments, 1000 ml resin condensers were used. The electrochemical measurements were performed in situ during the whole immersion period. The electrolyte used in this study was made of 0.6 M KOH + 0.2 M NaOH + 0.03 M Saturated Ca(OH)_2_ (pH = 13.3), which simulated the typical concrete pore solution [19]. NaCl was added into the electrolyte solution progressively until a concentration of 5 M. The time between two consecutive chloride additions was 24 h. The immersion time of rebar was 10 days. The solutions were replaced every 48 h, and fresh test solution was used for each period. Three replicates were used for each test.

### 2.2. Electrochemical Measurements

All the electrochemical tests were performed in a conventional three‑electrode cell where the sample of 1 cm^2^ area was exposed to the pore solution as the working electrode. A saturated calomel electrode (SCE) acting as the reference electrode was placed between the sample and the platinum plate. All potentials reported in this study were versus SCE. All the electrochemical measurements were monitored by a Princeton Parstat P4000 electrochemical system.

The open circuit potential (OCP) monitoring was performed prior to the other measurements. Then the electrochemical impedance spectroscopy (EIS) tests were performed after 1 h, 3 h, 12 h, 24 h, 4 days, 7 days, and 10 days using an applied AC signal amplitude of 10 mV RMS (root-mean-square) which showed no signs of non‑linearity. The frequency of EIS ranged from 100 kHz to 0.01 Hz. The fitting of EIS was done by using the ZSimpWin software (EChem Software, Ann Arbor, MI, USA). 

Following the EIS was the linear polarization resistance measurement. The sinusoidal potential perturbation was 10 mV vs. OCP. The scanning rate was 0.16 mV·s^−1^. 

In order to investigate the electronic property of the SS steel passive film, the Mott‑Schottky measurement was carried out at a fixed frequency of 1000 Hz in the potential range from 0.5 V to 1.0 V in the negative direction at a rate of 50 mV·s^−1^. A high scanning rate was used to reduce the influence of electroreduction of the passive film as well as changes in film thickness during the measurements. At a sufficiently high scanning rate, the defect structure within the passive film was “frozen-in”, which avoided the defect density from being affected by the potential [20,21].

### 2.3. Surface Analysis 

X-ray photo-electron spectroscopy (XPS, ULVAC-PHI Inc., Chigasaki, Japan) was used to determine the composition distribution along the longitudinal direction of the passive film in different conditions. The XPS measurements were carried out on samples after short-term passivation (1 day), long-term passivation (10-day), and 30-day chloride induction. 

The X-ray diffraction analysis was performed with a PHI Quantera SXM XPS system (ULVAC-PHI Inc.), using a monochromatized Al K X-ray source. The detecting area was around 3 μm in diameter. Sputter depth profiles were measured for the passive film of specimens using an argon gun with an Ar^+^ ion beam energy of 500 V and a beam current of 20 mA. A survey spectrum was recorded to identify the elements and high‑resolution spectra of the following regions were recorded: oxygen (O 1s), carbon (C 1s), chromium (Cr 2p), and iron (Fe 2p). Curve fitting was performed using the commercial software package XPSPEAK version 4.1, which contained the Shirley background subtraction and Gaussian-Lorentzian tail function.

The cross section of the passive film after 10-day passivation and 30-day chloride induction was examined by STEM (scanning transmission electron microscopy)/FIB (Focused Ion Beam). The STEM (TecnaiG220, FEI, Hillsboro, OR, USA) sample was prepared using a focused ion beam (FIB) system at an accelerating voltage of 30 kV, where a carbon deposition was used to protect the specimen surface [22,23]. Transmission electron microscopy (TEM), high‑angle annular dark-field scanning transmission electron microscopy (HAADF-STEM, TecnaiG220, FEI), and STEM energy-dispersive X-ray spectroscopy (HAADF-STEM-EDS) were conducted on an FEI Tecnai F20 transmission electron microscope at an acceleration voltage of 200 kV. The EDS (Energy Dispersive Spectrometer) analysis was performed in scanning transmission electron microscopy (STEM) mode using a probe diameter of 1–2 nm.

The surface morphology of the specimens after short‑period passivation of 1 h and 3 h was examined with AFM (Atomic Force Microscope, FEI). All of the AFM experiments were carried out in tapping mode using a cantilever with linear tips. The scanning area in the images was 200 nm × 200 nm and 5 μm × 5 μm, respectively.

## 3. Results 

### 3.1. Electrochemical Results

#### 3.1.1. Open Circuit Potential (OCP) and Linear Polarization Resistance (LPR) Measurements

**Figure 1:**
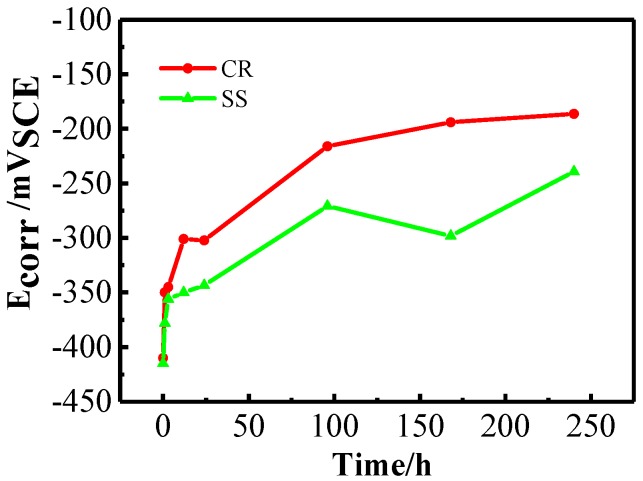
Corrosion potential (*E_corr_*) versus time behavior of CR and SS in simulated concrete pore solution.

**Figure 2:**
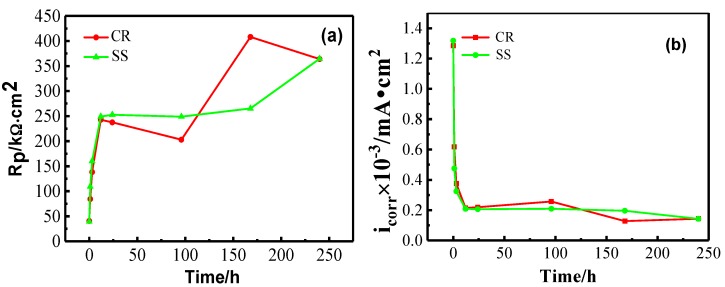
(**a**) Linear polarization resistance (*R_p_*); (**b**) corrosion current density (*i_corr_*) versus time behavior of steels in simulated concrete pore solution.

Figure 1 shows the comparison of the changes in the corrosion potential (*E_corr_*) of CR and SS steels measured in the simulated concrete solution. The values of *E_corr_* for CR and SS increase sharply in the first 24-h immersion. Thereafter, *E_corr_* increases continuously with a slower rate. A similar trend can be observed for the linear polarization resistance (*R_p_*) and the corrosion current density (*i_corr_*), as presented in Figure 2a,b. After 24‑h immersion, *R_p_* increases to 250 kΩ∙cm^2^ and *i_corr_* decreases to 0.2 mA∙cm^−2^, which reaches the standard of the protective passive film [24]. In other words, the formation of the passive film primarily occurs during the first 24-h passivation.

#### 3.1.2. Electrochemical Impedance Spectroscopy (EIS) Measurements

**Figure 3:**
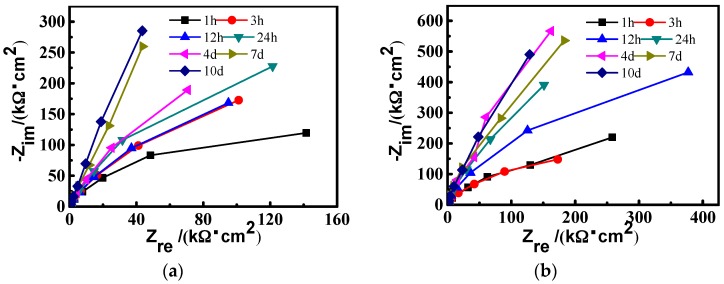
Nyquist plots of the EIS response of (**a**) CR steel and (**b**) SS steel in simulated concrete pore solution.

Figure 3 presents the time evolution of the Nyquist results of CR and SS in solution. The overall impedance of CR increases with time, which suggests a passivation process during the immersion period. An identical trend can be found for SS. It should be noted that the impedance change rate of CR is larger than that of SS in the first 3-h immersion, which indicates the larger change in the passive film of CR steel.

**Figure 4:**
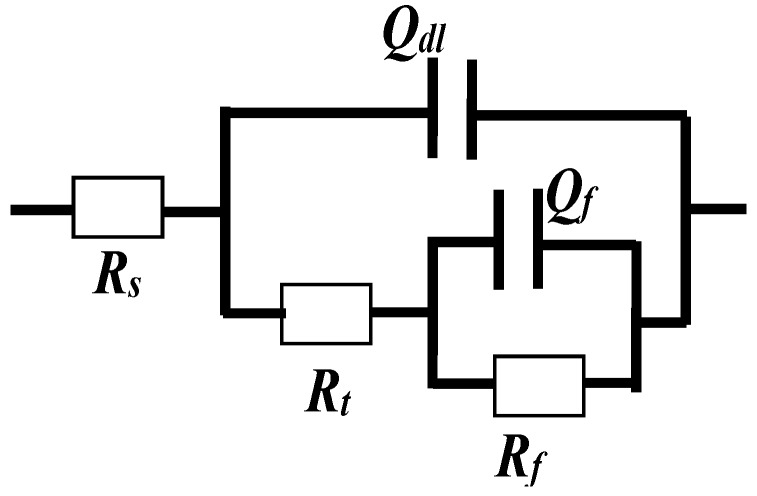
Equivalent circuit proposed to fit the experimental EIS data.

The corrosion resistance is quantified by means of the equivalent circuit which can be fit from the EIS results [25,26] as shown in Figure 4. The fitting parameters reflect the specific aspects of the protective ability of steel. For the present work, the following interpretation is proposed: The high frequency time constant (*R_t_*, *Q_dl_*) is represented by the charge transfer resistance (*R_t_*) and the admittance associated with the double layer capacitance (*Q_dl_*). The low frequency time constant (*R_f_*, *Q_f_*) is represented by the passive layer resistance (*R_f_*) and the passive film admittance (*Q_f_*). Due to the heterogeneity of the interface, *Q_dl_*and *Q_f_* are constant phase elements (CPE), of which the impedance is defined by Equation (1) [23].

(1) $$Z_{CPE}=1/Q{\left(j\omega \right)}^{\alpha }$$

where *Z_CPE_* means the impedance of the constant phase element, Q is defined as CPE, and the interpretation of this element depends on the α value.

Table 2 shows the comparison of the electrochemical parameters of CR and SS after long‑term passivation. The parameters, i.e., *R_ct_, C_dl_*, and *n* of CR steel are all similar to those of SS. In other words, the passive films of CR and SS have similar charge transfer resistance and roughness and thus similar corrosion resistance.

### 3.2. X-ray Photoelectron Spectrometer (XPS) Depth Profiling

**Figure 5:**
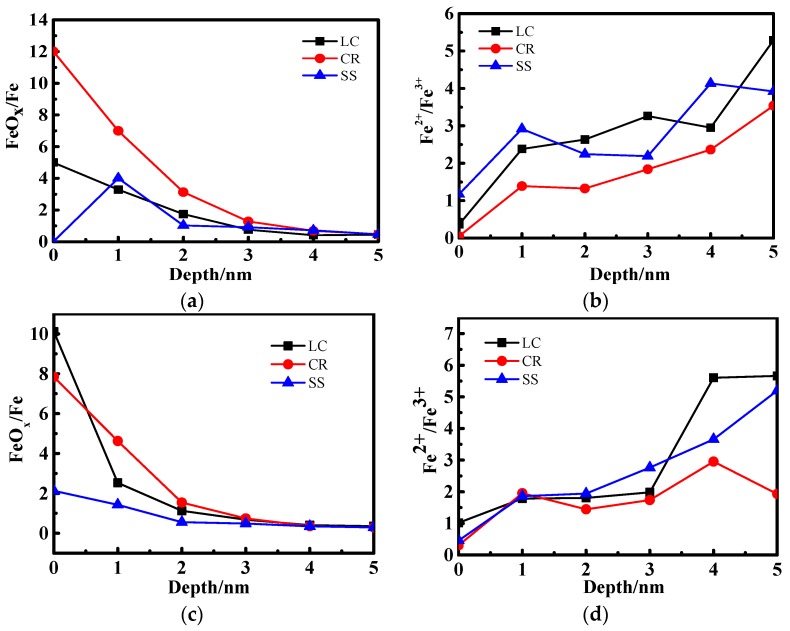
Comparison of the FeO_x_/Fe and Fe^2+^/Fe^3+^ for CR and SS at different passive film depths: (**a**) FeO_x_/Fe for 1-day immersion; (**b**) Fe^2+^/Fe^3+^ for 1-day immersion; (**c**) FeO_x_/Fe for 10-day immersion; (**d**) Fe^2+^/Fe^3+^ for 10-day immersion.

Figure 5 shows the iron oxide contents of CR and SS in different passive film depths after 1 day and 10 days. Figure 5a,c indicate that the iron oxide contents of CR and SS decrease with passive film depth and are the same after the 10‑day immersion. Figure 5b,d indicate that the content of Fe^2+^ in the passive film of CR is lower than that of SS regardless of the passive film depth.

**Figure 6:**
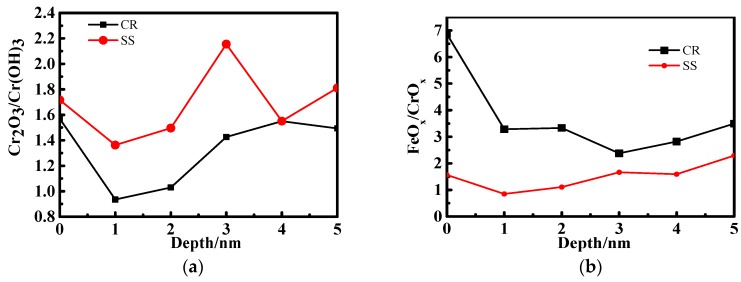

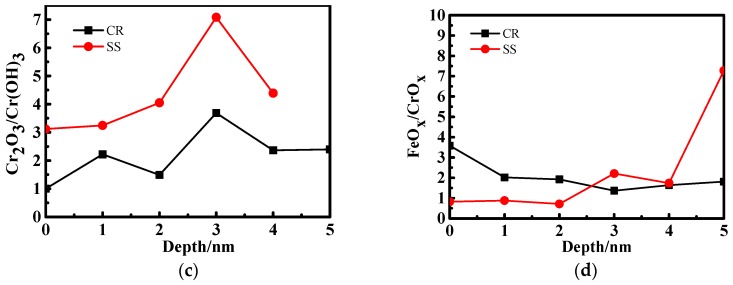
Comparison of the Cr_2_O_3_/Cr(OH)_3_ and CrO_x_/FeO_x_ for CR and SS at different passive film depths: (**a**) Cr_2_O_3_/Cr(OH)_3_ for 1-day immersion; (**b**) CrO_x_/FeO_x_ for 1-day immersion; (**c**) Cr_2_O_3_/Cr(OH)_3_ for 10-day immersion; (**d**) CrO_x_/ FeO_x_ for 10‑day immersion.

The chromium oxides in the passive film consist of Cr_2_O_3_ and Cr(OH)_3_. As shown in Figure 6a,c, the relative amount of Cr_2_O_3_ in the CR passive film is lower than that in SS and increases with the immersion time. Compared to the 1-day immersion shown in Figure 6b, the chromium oxide content of CR increases after the 10-day immersion shown in Figure 6d. Moreover, the chromium oxide content of CR exceeds that of SS when the passive film is deeper than 3 nm after the 10‑day immersion.

### 3.3. TEM-Longitudinal Section Image Analysis 

**Figure 7:**
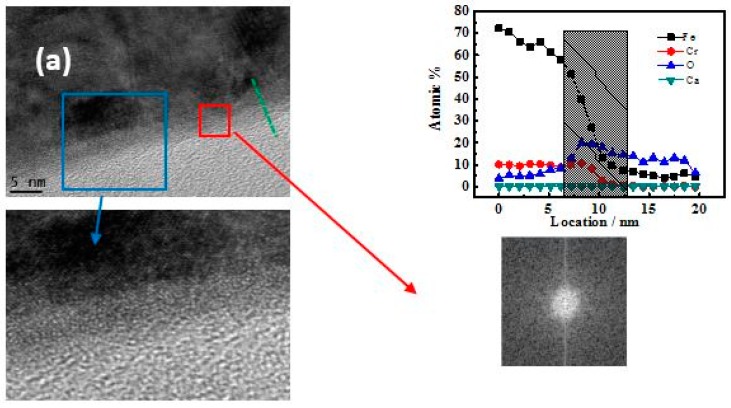

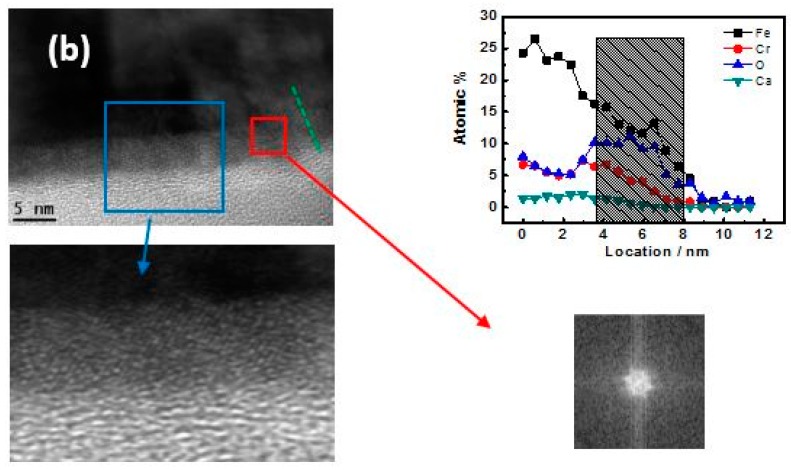
TEM morphology of passive films of CR and SS specimens after 10‑day immersion: (**a**) SS; (**b**) CR steel. Bright field image, located magnification image, atomic distribution, and FFT image.

Figure 7 shows the TEM bright field images of CR and SS after 10 day immersion. A thin oxide film is visible between the steel substrate and the glue layer in the TEM samples. The atomic distribution shows variation in the longitudinal direction of the passive film with respect to the content of the O element. In other words, the passive film exists. The oxide films have uniform thicknesses of around 5 nm and 3 nm for CR and SS, respectively. The lattice structure of the inner passive film of CR is consistent with steel substrate, which suggests an epitaxial growth model. In contrast, the lattice structure of the passive film of SS highly differs from the steel substrate.

The fast Fourier transform (FFT) images of the passive film region shows no diffraction contrast for the passive film of SS, while some white dots are detected in the FFT images of CR, indicating that the passive film of CR is semi‑crystalline while that of SS is amorphous.

### 3.4. Mott-Schottky (M-S) Analysis

The Mott-Schottky method assumes that the capacitance (C) of a semiconductor (passivated electrode) under depletion conditions depends on the applied potential (E) as follows,

(2) $$\frac{1}{C^{2}}=\frac{2}{\varepsilon \varepsilon_{0}qN_{q}}\left(E-E_{fb}-\frac{kT}{q}\right)$$

(3) $$N_{q}=\frac{2}{\varepsilon \varepsilon_{0}qS}$$

where *N_q_* is the carrier concentration (donor or acceptor), ε is the dielectric constant of the passive film, the relative dielectric constant of chromium oxides and iron oxides is 12 [27], *ε*_0_ is the vacuum permittivity, *q* is the elementary charge (−e for electrons and +e for holes), k is the Boltzmann constant, T is the temperature, U_fb_ is the flat band potential, and S is the slope of the M‑S curve.

**Figure 8:**
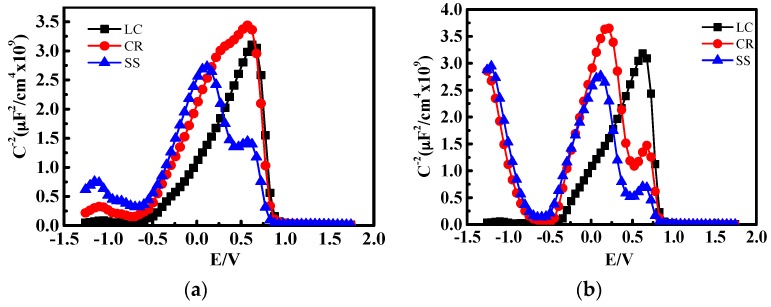
M‑S curve of CR and SS specimens after (**a**) 1-h and (**b**) 10-day immersion.

Figure 8 shows the Mott-Schottky plots for the films after 1-h and 10-day immersion. There are three parts in the M-S curves, i.e., −0.5 V–0.0 V, 0.0 V–0.5 V, and 0.5 V–0.75 V. For SS steel, there is a positive slope in the range of −0.5 V–0.0 V and 0.5 V–0.75 V, and a negative slope in the range of 0.0 V–0.5 V. This indicates a p-n semiconductor behavior. In contrast, only the positive slope exists for CR steel, which suggests an n-type semiconductor behavior. It should be noted that the slope value is larger than that in −0.5 V–0.0 V. This deep level is associated with the presence of Fe (II) ions and a few Cr (III) in octahedral positions [28].

**Figure 9:**
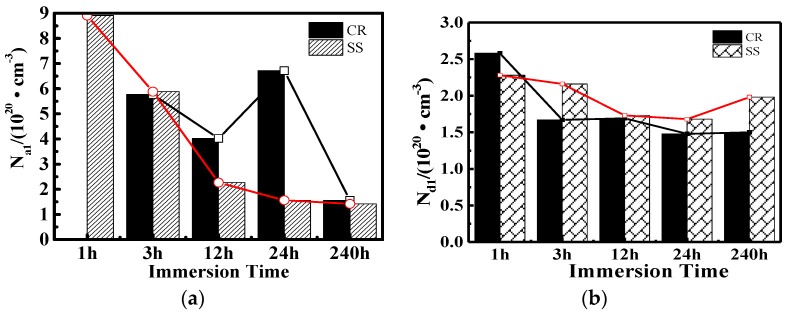
Carrier concentration development of CR and SS specimens with immersion time: (**a**) Acceptor carrier concentration *(N_a_*); (**b**) donor carrier concentration (*N_d_*).

According to Equations (2) and (3), the slopes are related to the donor (*N_d_*) and the acceptor (*N_a_*) densities. Figure 9 shows the carrier concentration development of the CR and SS specimens with immersion time. *N_a_* of CR varies significantly. No acceptor appears after the 1‑h immersion while eventually after 10-day immersion the value of *N_a_* reaches 2 × 10^2^ cm^−3^. By contrast, *N_d_* varies moderately from 2.5 × 10^2^ cm^−3^ to 1.5 × 10^2^ cm^−3^. A similar trend can be detected for SS.

In addition, it should be noted that *N_a_* for SS varies slightly, while that for CR decreases continuously after the 1-day immersion. As a result, after the 10‑day immersion, *N_a_* for CR is slightly higher than that for SS in the inner layer.

### 3.5. AFM-Cross Section Image Analysis 

**Figure 10:**
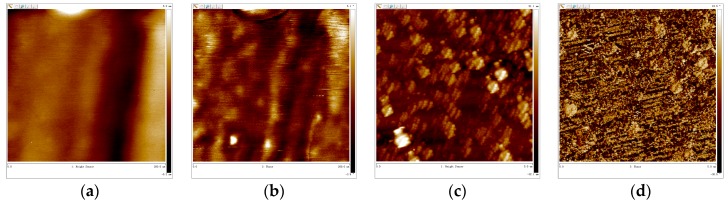

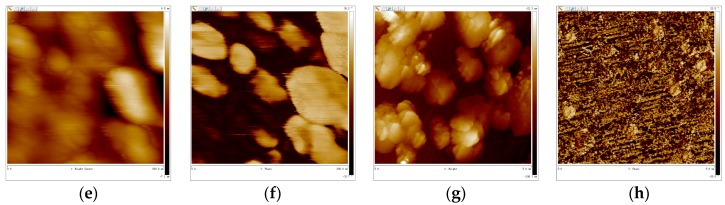
Surface morphology of CR and SS specimens during different passive times: (**a**) height image of the CR sample with 1-h immersion (200 nm × 200 nm); (**b**) phase image of the CR sample with 1-h immersion (200 nm × 200 nm); (**c**) height image of the CR sample with 24-h immersion (5 µm × 5 µm); (**d**) phase image of the CR sample with 24-h immersion (5 µm × 5 µm); (**e**) height image of the SS sample with 1-h immersion(200 nm × 200 nm); (**f**) phase image of the SS sample with 1-h immersion(200 nm × 200 nm); (**g**) height image of the SS sample with 24-h immersion (5 µm × 5 µm); (**h**) phase image of the SS sample with 24‑h immersion (5 µm × 5 µm).

Figure 10 shows the topographic data obtained from AFM. For the films formed on CR after the 1-h immersion, the topographic and phase images from Figure 10a,b indicate two kinds of small passive particles on the surface, the diameter of which is about several nanometers. Likewise, two kinds of passive particles grow on the SS surface as well, shown in Figure 10e,f, while the size is much larger than that on the CR. The particle diameters precipitated on the SS steel surface are dozens of nanometers, as shown in Figure 10c. The light part in the phase image could be the elastic Fe^3+^ hydrated oxide [29].

After 24-h immersion, the topographic and phase images of CR and SS reveal a dominant phase together with some localized regions (dark regions in Figure 10c,d,g,h). This suggests the formation of the passive layer and the existence of a different inner phase.

## 4. Discussion

### 4.1. The Effect of the Cr Element

The passive film growth process determines the eventual property of the passive film. The growth process varies with alkalinity, temperature, chloride, steel composition, etc. [30]. Thermodynamically, the steel with 13% Cr or above could form a stable protective passive film spontaneously in air [10]. Due to the low Cr content of 10% in the CR steel, a Cr_2_O_3_ protective film could not form on the steel surface in atmosphere spontaneously. The formation of Cr oxide on the CR steel surface relies on the high alkaline environment. A thin oxide layer forms after the 1-h immersion in solution according to Figure 8a, which behaves as an n-type semi-conductor. Generally, Fe(III) turns out to be n-type and Cr(III) oxide turns out to be p-type [31,32]. This indicates that it is mainly Fe rather than Cr that dissolves and precipitates during the initial passivation period.

After the 3-h passivation, a p-n semiconductor forms on the CR steel surface, indicating the appearance of Cr oxide behaving as a p-type semiconductor. The epitaxial growth of the CR steel inner passive film layer, of which the lattice structure is consistent with the steel substrate shown in Figure 6b, suggests the in situ reaction of inner passive layer. Due to the low content of Cr in the CR steel substrate, the slow dissolution of Cr^3+^ leads to the reaction with OH^−^ which arrives at the steel surface [33], eventually resulting in the in situ grown inner layer. The state of the passive film is influenced by composition and solution. According to the literature, for high Cr‑content steel, the faster dissolution rate of Cr leads to a dissolution-precipitation inner layer. The lattice structures of the Cr-enriched inner layer are crystalized or amorphous which is of a different lattice structure from the steel substrate [34]. Alloys with a high Cr content, such as Fe–20Cr–*x*Ni [34] and the 304 stainless steel [22], formed an amorphous film. In comparison, the alloy 690 formed a spinel oxide layer in high-temperature alkaline environment [29].

In the later passivation stage, the Cr oxide content in the SS passive film layer varies slightly due to inhabitation of OH^−^ and Cr transport through the passive film, resulting from the blocking of the compact Cr-enriched inner layer. By contrast, the Cr oxide content increases in the CR passive film in the later passivation period. Since little iron oxide could transport through the compact inner layer, the iron oxide content in the passive film changes slightly in the later passivation period as presented in Figure 5b,d. The initial passive film particles are of small size as shown in Figure 10b,c, and thus boundaries appear between the particles as a short‑circuit passage for OH^−^ to transport through, which react with Cr in the steel substrate in the late passivation period [35]. This benefits the formation of Cr oxide in the CR passive layer, which leads to the increase of chromium oxide in the later passivation period.

### 4.2. The Relationship Between Passive Film Structure and Electrochemical Property 

The electrochemical property directly reflects the passivity and corrosion resistance of the passive film. It is inherently determined by the passive film structure including the electronic and lattice structure.

After a 3‑h immersion, the passive film impedance of CR steel increases rapidly, as shown in Figure 3. This implies that the semiconductor might transform from n-type to p-n type. As a result of the carrier concentration gradient and the drift of the carrier at the interface, a stable space charge layer of much lower carrier concentration forms between p-type and n-type, which leads to the remarkable decrease of the passive film resistance for CR. 

The concentration of the acceptor carrier gradually decreases with passivation time due to the formation of Cr_2_O_3_, which leads to the decrease of cation vacancies. After long-term passivation, the concentration of the acceptor carrier of CR steel is equal to that of SS. Therefore, the passive film resistance *R_f_* of CR steel is similar to SS steel, as shown in Table 2.

The corrosion resistance of steel is also related to the passive film lattice structure. Harmful chloride ions could attack the passive film via voids or grain boundaries in the outer passive film layer [36]. Therefore, crystalized and amorphous passive films are both possibly vulnerable to the attack of chloride. The key point to avoid the attack of chloride is to reduce the defects in the passive film. For the high Cr‑content steel, the Cr^3+^ ions dissolve and precipitate on the steel surface and thus the Cr oxides are amorphous. Although ions slowly heal the defects during the long term passivation, defects still exist in the amorphous structure which are the result of the rapid reaction. The Cr content of CR steel is around half of that of the SS steel. The inner layer of the passive film of the CR steel is crystalized and the lattice structure is consistent with the steel substrate, as shown in Figure 7b. In other words, an in situ oxidation process takes place in the inner oxide layer, leading to the slow reaction rate and formation of oxide with less defects in the inner layer, contributing to the higher corrosion resistance.

### 4.3. Growth Mechanism of the Passive Film 

**Figure 11:**
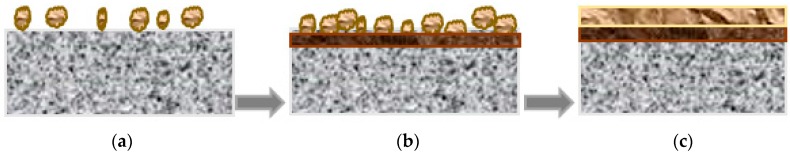
Schematic diagrams of the growth mechanism of the passive films on CR steel in a high alkaline simulated concrete solution.

A growth mechanism is proposed for the passive film of CR in the high alkaline simulated concrete pore solution, as schematically shown in Figure 10. The OH^−^ from the solution reacts firstly with selectively dissolved Fe in the outer passive film layer [37]. Plenty of nucleation sites lead to the precipitation of iron oxides and the formation of fine passive particles [38], as shown in Figure 11a.

The main reactions are described by Equations (4) and (5) [37].

Fe^2+^+2OH^−^→Fe(OH)_2_↓ (4)

Fe(OH)_2_+1/2O_2_+H_2_O→Fe(OH)_3_ (5)

Since the outer layer inhibits the direct contact between the dissolved Fe and OH^−^, after the initial formation of the outer layer, the remaining OH^−^ from the solution could penetrate into the outer layer and react with the dissolved Cr in the steel substrate.

The in situ Fe-Cr oxide inner layer then forms, as described by Equation (6) [22]. The semiconductor then transforms from n-type to p-n type [39,40]. The coalescence of the oxide islands leads to a roughly continuous oxide layer with defects, as shown in Figure 11b.

Cr^3+^+3OH^−^→Cr(OH)_3_↓ (6)

A continuous oxide layer could block the growth of the passive film [29]. The relatively low Cr content in CR passive film and a large number of particle boundaries facilitate the transport of Cr through passive film. This contributes to the further growth of passive film of CR steel. Since the continuous layer hinders the exposure of the inner layer to H_2_O in the solution, Cr(OH)_3_ converts rapidly into Cr_2_O_3_, as described in Equation (7). After long-time passivation, a stable passive film forms on the CR steel surface with a dual-layer structure, as shown in Figure 11c. The amorphous outer layer consists of Fe_2_O_3_ and FeOOH_3_. The crystalline inner layer is FeO‑Cr_2_O_3_.

2Cr(OH)_3_→Cr_2_O_3_+3H_2_O (7)

## 5. Conclusions

Due to relatively low 10% Cr content, the passivation of CR steel depends on the high alkalinity and the Cr-enriched inner layer grows from the steel substrate.

Due to the plenty of grain boundaries between the fine outer passive layer particles on CR steel as short-passages for OH^−^ transport, the Cr oxide content of the CR passive film increases continuously in the later passivation period.

The filling of Cr in the cation vacancies in the inner layer in the later passivation stage as well as the directly observed in situ growth model of the inner layer leads to a more compact and less conductive passive film.

A growth model is proposed for the CR steel in simulated concrete solution. The formation of the outer passive film layer is based on a dissolution-precipitation model, while the formation of the inner layer is based on an in situ growth model. Cr oxide still forms continually in the later passivation stage.

## Figures and Tables

**Table 1:** Chemical composition of samples (wt %).

Steel	C	Si	Mn	Cr	Cu	Ni	Al	Mo
LC	0.22	0.53	1.44	-	-	-	-	-
CR	0.01	0.487	1.49	10.36	-	-	-	1.162
SS	0.02	0.48	1.09	23.31	-	4.35	-	0.42

**Table 2:** Protective property comparison based on different electrochemical parameters.

Parameters	CR	Fitting Error	SS	Fitting Error
*R_ct_* (kΩ·cm^2^)	848,000	0.00%	903,000	0.00%
*R_f_* (kΩ·cm^2^)	1,210,000	0.00%	1,930,000	0.00%
*C_dl_* × 10^−5^ (μF·cm^−2^)	2.25	12.60%	2.77	26.10%
n	0.9756	34.95%	0.9411	66.13%
